# ENGAGING WITH NURSING HOME SOCIAL WORKERS TO DEVELOP AND IMPLEMENT CLINICAL INTERVENTION TRAINING

**DOI:** 10.1093/geroni/igad104.0156

**Published:** 2023-12-21

**Authors:** Katherine Supiano, Troy Andersen, Bradbury Laura, Kimberly Ponce Gonzalez

**Affiliations:** University of Utah, Salt Lake City, Utah, United States; University of Utah College of Social work, Salt Lake City, Utah, United States; University of Utah College of Nursing, Salt Lake City, Utah, United States; University of Utah College of Nursing, Salt Lake city, Utah, United States

## Abstract

PreLoss Group Support (PLGS) is a 10-session multi-modal therapeutic group intervention for dementia family care partners administered by nursing home social workers (NHSWs) to facilitate healthy death preparedness and eventual bereavement. In prior work, we used empirically validated procedures for developing psychosocial interventions, created PLGS participant and facilitator manuals, and implemented and evaluated PLGS in three cohorts of nursing home family care partners using research team LCSWs as facilitators and NHSWs as co-facilitator-trainees. Our present pragmatic trial will determine if telehealth-delivered PLGS reduces risk of poor bereavement outcome in family care partners, when delivered by NHSWs trained in PLGS via telehealth. We systematically engaged with nursing home stakeholders; administrators and NHSWs in rural, urban, and Veteran nursing homes to identify their perceptions of family care partner needs with respect to end-of-life care in residents living with dementia.NHSWs collaborated with our team to develop a full-day clinical education program. We incorporated their suggestions of family “characters” to script into our video modules using professional actors, addressed potential concerns with telehealth delivery of the training and intervention, participant identification and recruitment procedures, and requests for didactic content and interactive case examples of treatment element application. Current NHSWs who attended the full day training completed a 7-item satisfaction scale yielding a satisfaction rate of 95% (highly valuable), with 100% indicating they would recommend training to SWs in other facilities. Sustained collaboration with NH stakeholders optimizes commitment of NHSWs to PLGS participants, intervention delivery and informs later implementation strategies for translation and dissemination.

